# How Safe Is Safe Enough? Radiation Risk for a Human Mission to Mars

**DOI:** 10.1371/journal.pone.0074988

**Published:** 2013-10-16

**Authors:** Francis A. Cucinotta, Myung-Hee Y. Kim, Lori J. Chappell, Janice L. Huff

**Affiliations:** 1 NASA, Lyndon B. Johnson Space Center, Space Radiation Program, Houston, Texas, United States of America; 2 University of Nevada Las Vegas, Department of Health Physics and Diagnostic Sciences, Las Vegas, Nevada, United States of America; 3 Universities Space Research Association, Division of Space Life Sciences, Houston, Texas, United States of America; Albert Einstein College of Medicine, United States of America

## Abstract

Astronauts on a mission to Mars would be exposed for up to 3 years to galactic cosmic rays (GCR) — made up of high-energy protons and high charge (Z) and energy (E) (HZE) nuclei. GCR exposure rate increases about three times as spacecraft venture out of Earth orbit into deep space where protection of the Earth's magnetosphere and solid body are lost. NASA's radiation standard limits astronaut exposures to a 3% risk of exposure induced death (REID) at the upper 95% confidence interval (CI) of the risk estimate. Fatal cancer risk has been considered the dominant risk for GCR, however recent epidemiological analysis of radiation risks for circulatory diseases allow for predictions of REID for circulatory diseases to be included with cancer risk predictions for space missions. Using NASA's models of risks and uncertainties, we predicted that central estimates for radiation induced mortality and morbidity could exceed 5% and 10% with upper 95% CI near 10% and 20%, respectively for a Mars mission. Additional risks to the central nervous system (CNS) and qualitative differences in the biological effects of GCR compared to terrestrial radiation may significantly increase these estimates, and will require new knowledge to evaluate.

## Introduction

In space astronauts are exposed to galactic cosmic rays (GCR) comprised of high-energy protons and high charge (Z) and energy (E) (HZE) nuclei and solar particle events (SPE) comprised largely of low to medium energy protons. As space missions venture away from Earth into deep space, long-term exposures occur leading to important concerns about the risks to astronauts, including discussions on the acceptable risk level. A key component of this concern are the types of radiation that occur in space [1]–[6], which produce distinct types of biological damage from radiation on Earth such as X-rays or gamma-rays. Individual radiation sensitivity and estimating risks at low dose-rates are additional major concerns, while potential interactions between space radiation and microgravity found to be a minor concern [1]. Fatal cancer risk [1]–[6] has been considered the dominant risk for GCR and NASA has developed the NASA Space Cancer Risk (NSCR) model to estimate cancer risks and uncertainties for space missions [5], [6]. NASA's radiation standard limits astronaut exposures to a 3% risk of exposure induced death (REID) evaluated at the upper 95% confidence interval (CI) of the risk estimate [7]. However recent epidemiological analysis of radiation risks for circulatory diseases [8]–[10] shows additional risks, and allow for predictions of REID for circulatory diseases to be included with cancer risk predictions [5], [11], [12]. Risks to the central nervous system (CNS) are also a concern [1], [4], however methods to make quantitative risk estimates of CNS effects have not been developed.

Conjunction class Mars missions [13], [14], where Earth and Mars are in favorable alignments, involve long stays on the martian surface of approximately 540-d with transit times from Earth to Mars and back of about 400-d. Opposition class missions are more variable with launch date, whereby assuming a 60-d Mars surface time can lead to total transit times that vary from 460 to 780-d. In considering radiation risks, the impacts of solar modulation need to be included. GCR organ exposures vary by about 2-fold over the approximately 11-y solar cycle being highest at solar minimum when solar modulation of GCR is weakest [4], [5]. The frequency and size of solar particle events (SPEs) are difficult to predict, however their likelihood of occurrence decreases greatly for a 3-year period about solar minimum [5]. In this paper we make predictions near solar minimum for cancer and circulatory disease and discuss issues related to improving risk estimates and risk reduction for space missions. Predictions for SPEs will be considered in other reports.

## Methods

We used NASA's models of risks and uncertainties based on recent radio-epidemiology studies of cancer, GCR environmental models, particle transport codes describing the GCR modification by atomic and nuclear interactions in spacecraft and tissue shielding, and models of biological effectiveness of different radiation types [5], [6]. The model [5] includes NASA defined quality factors for solid cancer and leukemia risk estimates for HZE particles, and use of a never-smoker population to represent astronauts. Risk predictions were made for missions near solar minimum using the average of these derived from historical data on sunspot numbers for solar cycles 1 to 24, and fitted to modern data on GCR composition and energy spectra [5], [14], [15]. Transport codes describe the atomic and nuclear interactions of particles including projectile and target nuclei fragmentation and production of light particles (protons, neutrons, helium etc.) [16], [17]. Recent spacecraft such as the International Space Station (ISS) or Orion capsule developed as an exploration mission crew transfer vehicle have an average of about 20 g/cm^2^ equivalent aluminum shielding, which is used in risk calculations. For the martian surface we use an average shielding thickness of 10 g/cm^2^ to represent a light surface habitat, and included the martian atmosphere represented by CO_2_ with a 18 g/cm^2^ vertical height. Results for the ISS include the trapped protons [18] along with GCR. Previous reports demonstrate that NASA's model agrees with spaceflight dosimetry measurements [5], [18], [19] to within 15%. Organ doses and probability distribution functions (PDF) describing uncertainties in model parameters [5] are summarized in Tables S1 and S2 in File S1, respectively.

Circulatory disease risks included cardiovascular disease (CVD) and ischemic heart disease (IHD) using excess relative risk (ERR) estimates from a recent meta-analysis of studies of atomic bomb survivors, and nuclear workers in several countries [8]. Circulatory disease risk estimates were made using the non-cancer effects dose equivalent for the blood forming system (BFO) based on a distinct relative biological effectiveness (RBE) factor compared [20], [21] to that of cancer estimates, and without the use of a dose and dose-rate reduction effectiveness factor (DDREF). These choices and alternative ones are discussed below. Organ dose equivalents for cancer risk are given in units of Sievert (Sv). For circulatory disease risks because the RBE is distinct from the quality factor (QF), organ dose equivalents are expressed in terms of a different unit, Gray-Equivalent (Gy-Eq) [21]. A detailed description of REID models and uncertainty analysis using Monte-Carlo propagation of uncertainty is described in the Supplementary material and prior report [5].

## Results

**Figure 1:**
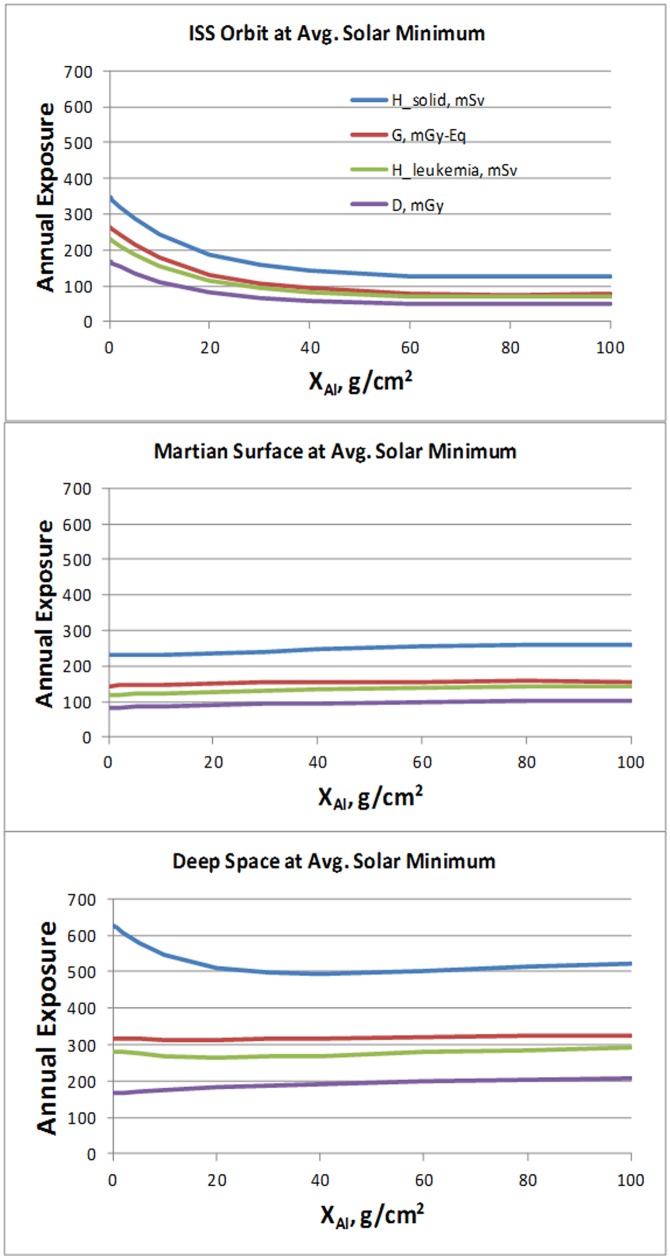
GCR organ dose characterization at average solar minimum. (A) The attenuation of annual GCR organ averaged doses versus depth of aluminum shielding for deep space, the martian surface, and for combined GCR and trapped protons in the ISS orbit. Calculations are for males absorbed dose (D) (mGy), solid cancer and leukemia risks (H) (mSv), and non-cancer effects (G) (mGy-Eq). Calculations on the martian surface consider its atmosphere with an 18 g/cm^2^ CO_2_ vertical height.

Calculations of tissue average absorbed doses, non-cancer risk dose equivalent [20], [21], and NASA dose equivalent for leukemia and solid cancer risks [5] were made for up to 100 g/cm^2^ of aluminum shielding (Fig. 1). GCR doses were not sensitive to shielding amounts due to a near balance in particle loss and production through atomic and nuclear interaction that occur within shielding. Organ specific doses also show small variation (Table S1 in File S1) due to the high energies of GCR and secondary radiation. For SPE's a much larger variation between doses at individual organs occurs [5].

**Figure 2:**
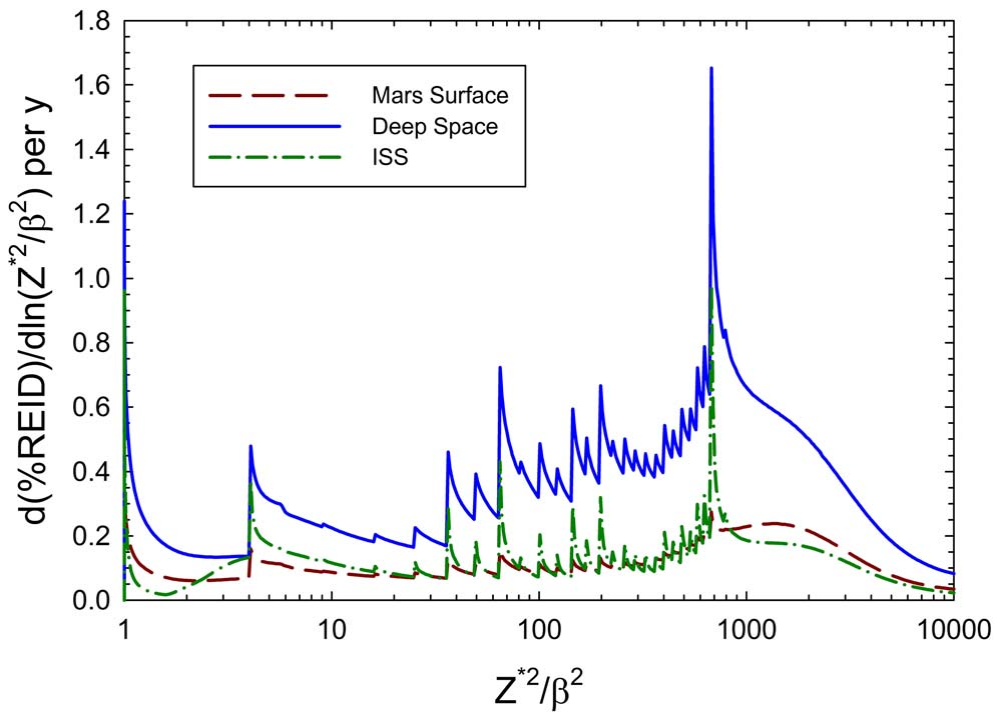
Distribution of %REID for solid cancer for particles represented by Z*^2^/β^2^ for deep space, martian surface and ISS orbit for 20 g/cm^2^ shield. The full GCR spectrum that traverse astronauts in deep space are more biologically damaging compared to the higher energy GCR that occur in low Earth orbit.

The distribution spectra of %REID per year for solid cancer versus the GCR descriptive parameter, Z^*2^/β^2^, where Z* is a particles effective charge number and β its velocity, is shown in Fig. 2 for average spacecraft shielding conditions. The parameter Z^*2^/β^2^ describes the density of the ionization of a particle track more effectively than LET and is used in the NASA quality factor [5]. A prominent peak occurs near 26^2^ corresponding to relativistic iron particles with similar peaks observed for other HZE particles. The contributions at small values of Z^*2^/β^2^, which have low biological effectiveness, are increased for ISS due to the trapped protons in the ISS orbit. The martian atmosphere provides some protection from HZE particles, however leads to a buildup of particles at small Z^*2^/β^2^ such as protons and pions, and an increased contribution from neutrons.

**Figure 3:**
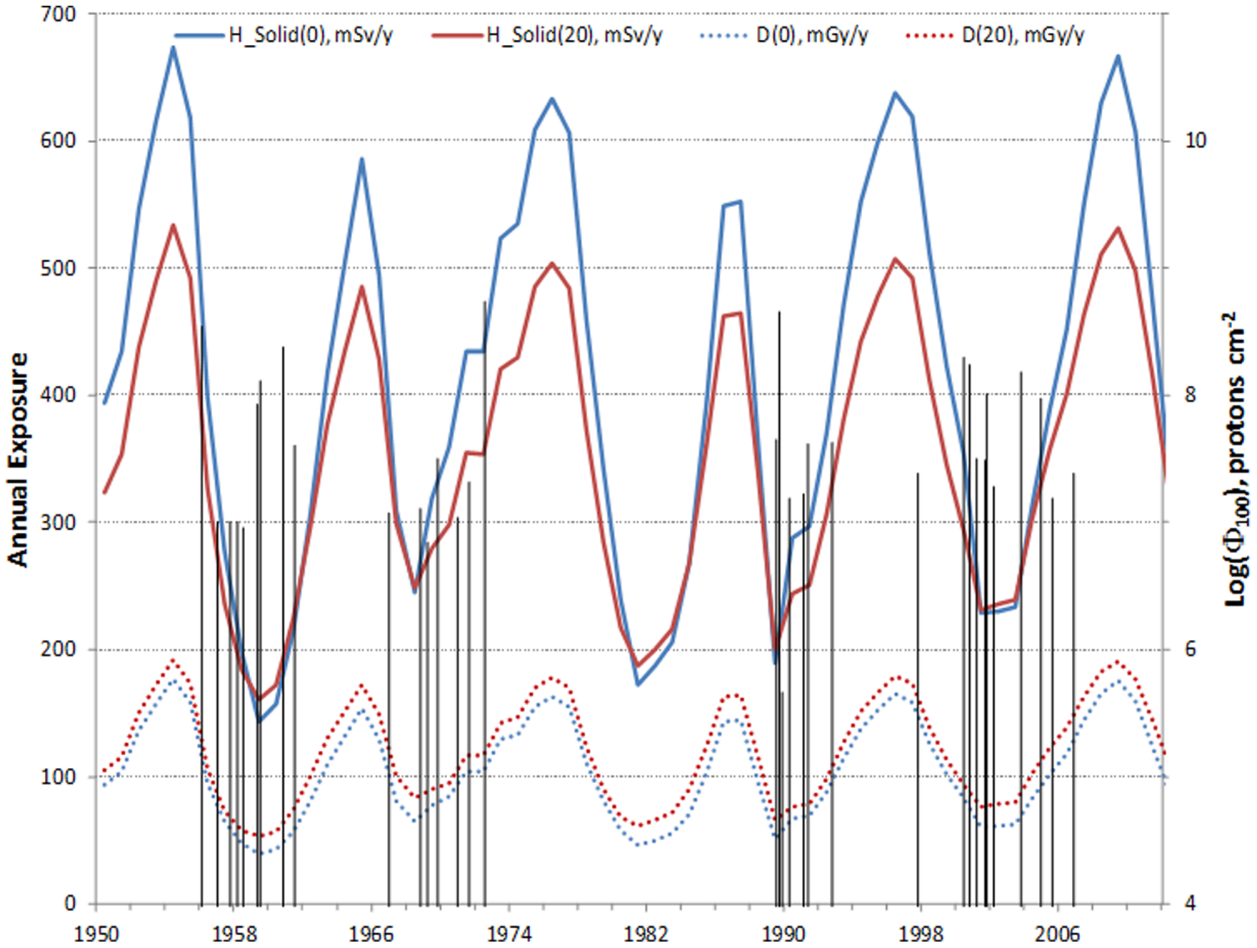
Estimates of the GCR organ doses over recent solar cycles at 0 and 20/cm^2^ of aluminum shielding (left axis) and the log of the 100 MeV integral proton fluence, which was shown to be a useful predictor of SPE organ doses after considering their variable energy spectra (*11*) (right axis).

Figure 3 shows predictions of the dependence of GCR absorbed dose and solid cancer dose over time from 1950 to 2012. Also shown are times for the 43 largest SPE's out of ∼400 observed since 1950, which corresponds [22] to a lower cutoff for the integral fluence of 100 MeV protons of >10^6^ p/cm^2^. SPEs with smaller values for 100 MeV integral fluence will have tissue doses below 0.01 Gy for light spacecraft shielding and are not shown. These results show the anti-correlation between GCR and SPE doses that will occur between solar minimum and maximum, respectively. Variation of GCR organ dose equivalents of up to 20% can occur when comparing different solar minimum.

**Figure 4:**
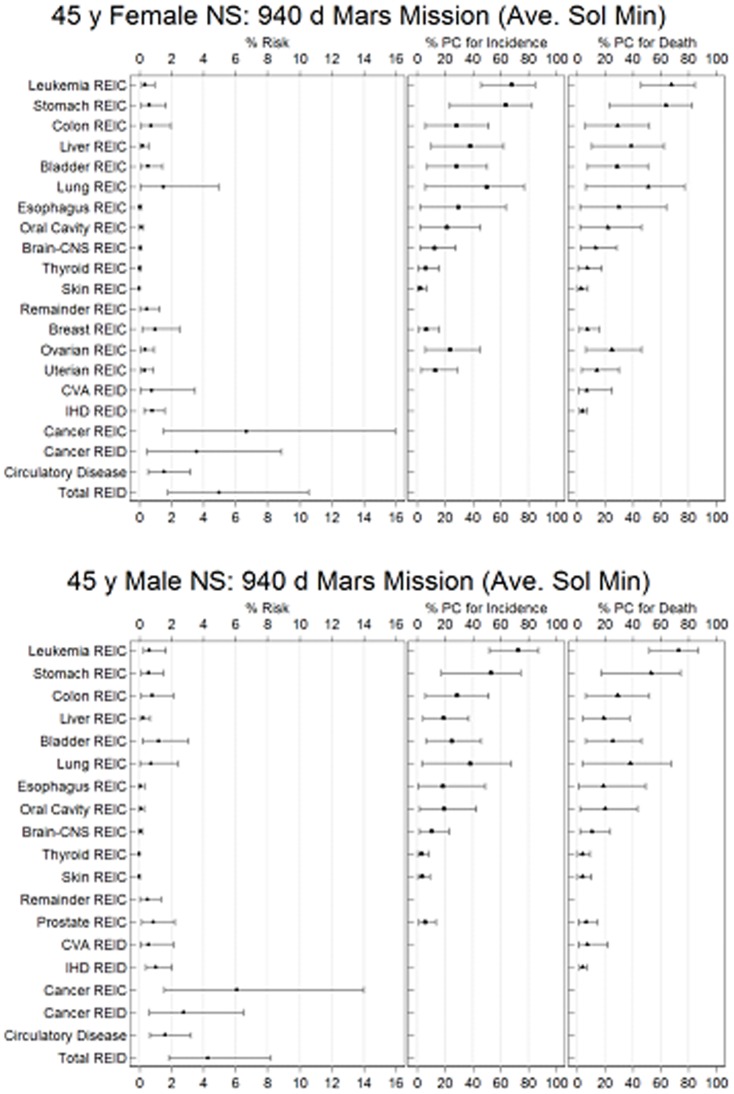
Estimates of tissue specific %REIC, %PC for incidence and death and %REIC and %REID for 940-d Mars mission with average solar minimum conditions. (A) Values are for an average 45-y female never smokers and (B) for an average 45-y male never smokers. Calculations assume 20 g/cm^2^ and 10 g/cm^2^ aluminum shielding for transit and martian surface, respectively. PC estimations are for 20 years post mission. All point assessments are bracketed by 95% confidence intervals.

We predicted tissue specific radiation-exposure incidence of cancer (%REIC), and %REID for overall cancer risks for 940-d Mars missions by 45-y old female and male never-smoker (NS) populations (Fig. 4) for heavy shielding. The circulatory disease risks for NS are similar to the U.S. average population (Table 1), which is largely due to NS's longer lifespan, whereby the radiation associated circulatory disease risk up to age 85-y is lower for NS compared to the U.S. population but is similar over all ages. The combined %REID exceeded NASA limits [7] by about 3-fold. Predictions for fatal cancer risk are about 25% higher for females compared to males. For combined cancer and circulatory disease fatal risk, females are about 15% higher risk compared to males. The added contributions to the %REID from circulatory diseases was predicted to increase %REID by about 40% and to reduce the age at exposure dependence on %REID compared to cancer risks alone.

**Table 1:** Lifetime risks for the 940

	*%REIC, Cancer*	*%REID, Cancer*	*%REID,Circulatory*	*% REID, Combined*
	45-y Females			
**U.S. Average**	9.15 [0.95, 22.2]	5.32 [0.95, 14.3]	1.48 [0.57, 3.05]	6.57 [1.38, 14.8]
**Never-Smokers**	6.66 [1.52, 16.0]	3.56 [0.51, 8.87]	1.55 [0.58, 3.20]	4.98 [1.77, 10.6]
	45-y Males			
**U.S. Average**	7.41 [1.79, 17.0]	3.52 [0.66, 8.23]	1.53 [0.64, 3.05]	4.94 [1.91, 9.78]
**Never-Smokers**	6.09 [1.56, 14.0]	2.75 [0.63, 6.52]	1.62 [0.68, 3.21]	4.28 [1.86, 8.22]

Comparison of lifetime risks for 45-y Females and Male for U.S. Average population and a population of never-smokers. The morbidity for circulatory diseases has not been directly estimated, but the addition of the %REIC (cancer) and %REID for circulatory diseases, indicate an upper 95% CI for the combined morbidity near 20%.

The probability of causation (PC) (also denoted as attributable risk) is a conditional probability used as an indicator of a potential causal relationship between radiation exposure and occurrence of disease in a population. Our predictions (Fig. 4) suggest that a large portion of cancers that would be observed in crews after exploration missions would be attributed to GCR exposure, with PC for leukemia, stomach, colon, lung, bladder, ovarian, and esophageal cancer significant. PC will increase modestly for longer post-mission times for most solid cancers and circulatory diseases, and decrease for leukemia. PC estimates for CVD and IHD were smaller than for many cancers because of the larger background occurrence for these diseases. Estimates for CVD and IHD incidence were not made, since only values ERR for mortality were available from the meta-analysis of Little et al. [8]. However obviously morbidity risks for circulatory diseases would be larger than mortality risk estimates and therefore add substantially to the overall morbidity of astronauts returning from a Mars mission.

**Figure 5:**
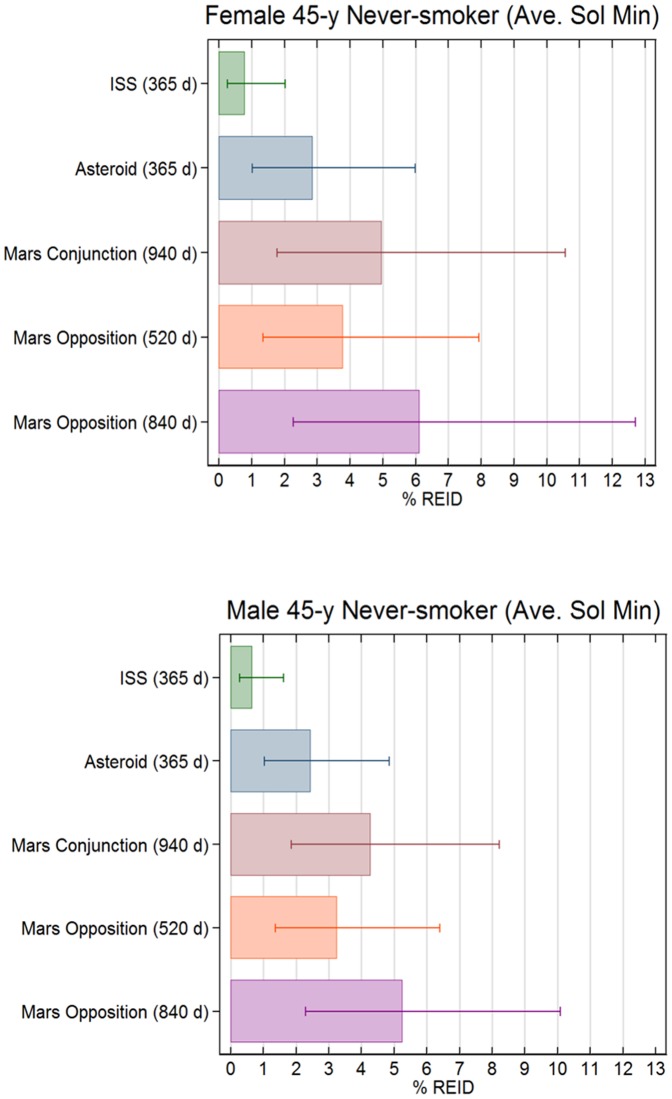
Comparison of %REID from cancer and circulatory diseases combined for several space exploration missions. (A) Estimates are shown for 45-y old female and (B) male never-smokers. Calculations assume 20 g/cm^2^ and 10 g/cm^2^ aluminum shielding for transit vehicle and martian surface habitat, respectively. Error bars reflect the 95% confidence intervals.

The %REID and %REIC for various space missions including 1-year on ISS, 1-year near- Earth asteroid (NEA) mission, and the Mars conjunction and opposition missions were predicted (Fig. 5). Risk was much less on ISS compared to deep space missions, and missions of 1-year on the ISS at solar minimum are within the acceptable risk level for astronauts [7]. In contrast, because the exposure is to all GCR energies and the longer mission duration, exploration missions exceeded NASA's radiation limit by a large amount. The upper 95% CI for the %REIC is estimated near 15% and the inclusion of other significant morbidity as described below, would increase this value to above 20% for returning crew. An improved situation occurs near solar maximum [4], [5] where GCR risks decreased about 2-fold (Fig. 3). At solar maximum there is the mission operations burden to respond to SPE's, which can occur every few months and are difficult to monitor from Earth when Mars is in opposition [4]. Also, the residual dose behind shielding from SPEs will increase REID by a variable amount depending on the SPE size and spectra, and mission operation responses including shielding availability.

Table 2 shows estimates of the maximum number days in space near solar minimum where NASA's limits are not exceeded for different ages at exposure and demographic variables. Results are for a specific shielding configuration of 20 g/cm^2^ aluminum, and include comparisons for fatal cancer risk alone or with the additional REID contributions from circulatory diseases. The addition of the circulatory disease risks reduces allowable times in space by two to three months depending on demographic considerations of crew composition. Variations of up to 10% would occur for other shielding designs (materials or additional shielding of a few 10′s of g/cm^2^). The uncertainties in the risk estimates are large, approaching a 3-fold ratio of the upper 95% CI to the central estimates. Reducing this uncertainty could substantially increase the number of days to stay within NASA's limits.

**Table 2:** Demographic Specific Solar Minimum Safe Days in deep space, which are defined as the maximum number of days with 95% CL to be below the NASA 3% REID limit for males and females at different ages at exposure, a_E_.

	a_E_, y	Cancer Risk		Combined Cancer and Circulatory Disease Risk	
		US Avg. Population	Never-smokers	US Avg. Population	Never-smokers
**Male**	35	209 (205)	271 (256)	173 (163)	208 (193)
	45	232 (227)	308 (291)	187 (173)	222 (214)
	55	274 (256)	351 (335)	206 (194)	256 (235)
**Female**	35	106 (95)	187 (180)	115 (107)	159 (149)
	45	139 (125)	227 (212)	123 (118)	183 (171)
	55	161 (159)	277 (246)	142 (129)	201 (191)

Calculations are for average solar minimum with 20 g/cm^2^ aluminum shielding. Values in parenthesis are the case of the deep solar minimum of 2009.

## Discussion

In this paper we made predictions of cancer and circulatory disease risks for space exploration missions to Mars near solar minimum using NASA's recent model developments [5], [6], and results from a recent epidemiological analysis [8] of circulatory disease risks from human exposures to low LET radiation. The combined risk was shown to increase %REID by about 40% from predictions of cancer risk alone. For circulatory disease predictions we used the deterministic effects RBE model recommended by the International Commission on Radiological Protection (ICRP) as our central estimate, which leads to a lower GCR organ averaged equivalent dose compared to solid cancer risk and higher value compared to leukemia risk. One assumption would be that the higher RBE from animal experiments for solid cancers would be the largest RBE that could be expected for circulatory disease risk. In our calculations the ratio for solid cancer to circulatory disease, GCR organ averaged dose equivalent varied between 1.7 and 1.9 for typical different spacecraft shielding amounts. Based on these observations, we used a log-normal distribution with geometric mean, GM = 1 and geometric standard deviation, GSD = 1.35 for the probability distribution function (PDF) representing the uncertainty in the tissue averaged RBE for circulatory disease (Supplementary material).

There are several areas where new information related to the current estimates could lead to reduced uncertainties and perhaps lower risk estimates. Of critical importance is understanding of DDREFs and RBEs for cancer and circulatory risks where data for many tissues are not available for HZE particles at relevant doses and dose-rates [4], [5]. Improved information on specifying tissue specific transfer weights used in applying epidemiology data, and understanding differences in disease rates between model populations would also reduce uncertainties in risk estimates.

Concerns about a possible dose threshold for circulatory disease risks, which is an important consideration for ISS missions [21], should be reduced for a Mars mission because organ doses are above where threshold doses have been estimated [9]. and because we used the meta-analysis results that were based in-part to chronic exposures of radiation workers [8]. For cancer risk predictions a dose and dose-rate reduction effectiveness factor (DDREF) of 1.5 is used for solid cancer estimates based on the BEIR VII report [12], and the recommendations of the National Research Council to NASA [6]. For circulatory disease risk predictions a DDREF is not applied because models were based on meta-analysis of several chronically exposed populations as described by Little et al. [8]. For cancer risks the low value for the DDREF of 1.5 leads to an uncertainty distribution that is skewed towards higher DDREF values and lower REID, which opposes the QF uncertainty estimate which is skewed to higher REID values [5].

The ERR models for circulatory disease do not include gender specific and time dependent factors because analysis suggests these modifiers are weak based on existing data [8]. This is in contrast to cancer risk estimates where more detailed models have been developed which include gender specific estimates, and consideration of age at exposure and time after exposure effects [11], [12]. In addition, cancer risk estimates consider both multiplicative and additive risk models, and uncertainty analysis considers choice for weighting these models [5], [6], [12], [23].

Lifestyle factors for circulatory diseases likely play a major role in considering radiation effects. However, the analysis made by current reports suggest that estimates of ERR do not vary significantly when adjustments for possible lifestyle factors are considered [8]–[10]. The NASA models for never-smokers [5] and US average populations did not lead to very different circulatory disease risks due to a cancellation of the combined effects of lower background rates for NS and their long life-span that leads to additional risks. Estimating uncertainties in radiation estimates due to healthy workers effects and lifestyle factors are important areas for future research.

Our predictions are incomplete in several aspects. First the qualitative differences in biological damage of HZE particles and secondary neutrons compared to low linear energy transfer (LET) radiation such as X rays or gamma-rays have not been addressed. Our calculations only consider the quantitative differences using quality factors based on experimental studies from particle accelerators simulating GCR components in mice and other small animals, and human cell culture studies. Qualitative differences originate in the much larger energy deposition and distinct spatial distributions of damage in biomolecules, cells and tissues by HZE particles compared to low LET radiation. HZE particles produce complex DNA damage leading to mutations with high frequency [5], [24], [25], and differences in the generation of reactive oxidative species (ROS) or free radicals. These differences result in higher levels of chronic oxidative stress and genomic instability [26].

Non-targeted effects (NTE) [26] include bystander effects, which occur in neighbor cells of damaged cells, and genomic instability, as a delayed effect in the progeny of the initial cells or tissues irradiated. NTEs can lead to non-linear responses at low dose (less than one HZE particle per cell) and higher relative biological effectiveness (RBE) values [27], which would lead to an overall increased cancer REID compared to current estimates. RBE's for tumor induction in mice for iron particles vary greatly with values near 30 obtained for Harderian gland tumors [28] and more than 50 for liver tumors [29]. Understanding possible mechanisms for tumor induction, including NTE's and genomic instability, would help improve approaches to select the RBE's to be used for human risk assessments. An additional concern is the potential earlier appearance and increased lethality of tumors induced by HZE particles and neutrons [28]–[31], which suggests an important qualitative difference not accounted for in current cancer risk estimates for space radiation.

A second area where risk estimates are incomplete is in the inclusion of risks from a range of additional radiation effects occurring at both early and late time points post exposure. These include added components to the circulatory disease risk profile such as increased likelihood for coronary revascularization and myocardial infarction [8]–[10]; risks for other degenerative or premature aging-related endpoints such as earlier appearing cataracts [32], musculoskeletal system effects including osteoporosis and exacerbation of microgravity associated loss of bone strength [33], and respiratory diseases [34].

An expanding body of evidence derived from ground based research using rodent models at particle accelerators that simulate GCR points to a potential risk for disruptions in cognitive performance and memory that may occur within the time scale of an exploration class mission impacting its success [35], [36]. These effects have been observed at low doses of HZE particles (<0.2 Gy) in the hippocampus, striatum and prefrontal cortex, which are correlated with molecular and cellular damage including persistent ROS, altered dopamine expression, apoptosis, neuroinflammation and altered neurogenesis [35]–[40]. A recent observation is acceleration of Alzheimer's disease (AD) pathologies following low dose iron particle exposures in transgenic mice [40]. The substantiation of excess risk for AD by GCR would further increase REID estimates from the current results, and introduce additional morbidity for returning crew.

An important debate surrounds acceptable risks for a Mars mission, which could have historical importance to civilization. Space missions are designed [41] to an aggregate risk for during mission loss of crew (LOC) of less than 1 in 270, with new technology investments expected to reduce LOC to less than 1 in 750. Actual occurrences have led to individual mortality of 0 or 1.6% for the ISS and all NASA programs [42], respectively. The average life-loss for an astronaut of 45-y age at exposure for a radiation induced cancer is estimated at about 15 years for gamma-rays [12], [13] and expected to be higher for HZE particles based on animal studies [28]–[31], or about 2.5 times less than an estimated ∼40 life-loss years for a during mission LOC. For circulatory disease risks, estimated life-loss is a few years smaller compared to solid cancers for low LET [8], [9], however not much is known for high LET radiation. Using the ratio for differences in average life-loss and considering a 4 to 6 person Mars mission crew size, suggests that a 1 to 10 ratio of during mission LOC design criteria to REID limit would be a comparable risk basis. On this basis, the 1 in 270 aggregate risk for during mission LOC is then quite similar to the current 1 in 33 radiation fatality limit at NASA [7], [21], while an aggregate risk goal of 1 in 750 recommended by the NASA Aerospace Safety Advisory Panel [41] would suggest a lower radiation limit should be a future goal for radiation protection. Other considerations are the additional radiation morbidity risk, and ethical considerations that value life at middle or old age, as opposed to considerations of LOC during the mission alone.

Economic investments to lower radiation risk could have great benefits on Earth in understanding low dose radiation risks after the Fukushima nuclear reactor accident in Japan, or concerns of the risks from diagnostic use of radiation such as CT-scans. Planning missions to avoid solar minimum may be the largest factor to decrease radiation risks (Fig. 3), however such plans could be overcome by mission timeline constraints and limitations in predicting future solar cycle characteristics. Other possible investments to mitigate risks include research on radiation shielding, genetic testing for selection crew at lower risk, and biological countermeasures. However, radiation shielding plays an important role for SPE protection but is not a solution to the GCR risk problem with current launch capabilities which limit shielding mass (Fig. 1). Water or hydro-carbon materials would only provide modest benefits of up to 10% compared to aluminum shielding for GCR [5], [42], [43].

Crew selection for individual radiation sensitivity and biological countermeasures (BCMs) potentially have the highest payoffs in reducing risks, however much more information is needed in these areas [4], [44], [45]. Selecting astronauts against radiation sensitivity [45] could be hampered by a small pool of astronauts and other constraints on crew selection. Of importance is that because population averaged values for overall mortality and morbidity risks could exceed 10% and 20% respectively, individuals of increased sensitivity could be especially vulnerable to GCR based on our current but limited knowledge on radiation sensitivity [44]. This observation should lead to requirements to test potential crew members for DNA damage repair capacity, ROS responses, and susceptibility to specific diseases, and investments in research to develop accurate approaches to do so.

Developing BCMs to GCR pose severe challenges due to the chronic exposure, and the need for protection against high LET radiation, which appear to act through distinct biological mechanisms [1]–[6]. It is not clear that BCMs developed to protect against acute radiation risks from high doses of low LET radiation will be helpful or harmful for GCR [45]. Acute risk BCMs often have unwanted side-effects, and work through the rescue of cells from apoptosis. For GCR such approaches could be untenable for long dietary intake of 940 d and could leave genetically unstable cells available to increase the risks of late effects. For both the development of BCMs and selection of astronauts, a severe challenge is the large number of diseases contributing to the overall risk. This suggests investments are best made in understanding the underlying biological mechanisms that could be applied to reduce uncertainties for many distinct risks. BCM efficacy must be established quantitatively with small uncertainty for GCR, which may be achieved through ground based research at particle accelerators [1]–[6]. Such research should allow for an operational decision to use such BCMs to enable missions projected to exceed risk limits, and would have many benefits on Earth including reducing the health effects from exposure to radiation.

## Supporting Information

File S1 **Contains: Supplementary Methods, Supplementary References, Table S1, Table S2.**
(DOCX)
