# Supplementary material for: How Safe Is Safe Enough? Radiation Risk for a Human Mission to Mars
Source: PLoS One. 2013 Oct 16;8(10):e74988. doi: 10.1371/journal.pone.0074988 (PMC3797711; doi:10.1371/journal.pone.0074988)
Supplement: File S1 — Contains: Supplementary Methods, Supplementary References, Table S1, Table S2. (DOCX) [file pone.0074988.s001.docx]

**Supplementary Methods**

The instantaneous cancer incidence or mortality rates, λ_I_ and λ_M_, respectively are modeled as functions of dose *D*, or dose-rate *D_r_*, gender, age at exposure *a_E_*, and attained age *a* or latency *L,* which is the time after exposure *L=a-a_E_*. The λ_I_ (or λ_M_ ) is a sum over rates for each tissue that contributes to risk, λ_IT_ . These dependencies vary for each cancer type that could be increased by radiation exposure. The total risk of exposure induced cancer (REIC) is calculated by folding the instantaneous radiation cancer incidence-rate with the probability of surviving to time *t,* which is given by the survival function *S_0_(t)* for the background population times the probability for radiation cancer death at previous time, and then integrating over the remainder of a lifetime:

$$\begin{matrix} (1) & REIC(a_{E},D) & = & \int_{a_{E}} dt\lambda_{I}(a,a_{E},D)S_{0}(t)e^{-\int_{a_{_{E}}}^{t} dz\lambda_{M}(z,a_{E},D)} & \end{matrix}$$

where z is the dummy integration variable. The Risk of Exposure Induced Death (REID) is similar to Eq.(1) with *λ*_I_ replaced by the mortality rate, *λ*_M_. After adjustment for low dose and dose-rates though the dose and dose-rate effectiveness factor (DDREF) and radiation quality, the tissue-specific, cancer incidence rate for an organ dose equivalent, *H_T_* , can be written as a weighted average of the multiplicative and additive transfer models, often called a mixture model:

$$\begin{matrix} (2) & \lambda_{IT}(a_{E},a,H_{T}) & = & [v_{T}ERR_{T}(a_{E},a)\lambda_{0IT}(a)+(1-v_{T})EAR_{T}(a_{E},a)]\frac{H_{T}}{DDREF} \end{matrix}$$

where *v_T_* is the tissue-specific transfer model weight, *λ_0IT_* is the tissue-specific cancer incidence rate in the reference population, and where *ERR_T_* and *EAR_T_* are the tissue specific excess relative risk and excess additive risk per Sievert, respectively. Equation (2) is summed overall tissues contributing to REID (or similarly to REID) to evaluate total cancer risk. The integration in Eq. (1) assumes a minimal latency of 2 years for leukemia and 5-years for cancer and circulatory diseases. The NASA Space Cancer Risk (NSCR)-2012 Model [5] uses the UNSCEAR report [11] fitted EAR and ERR models for most tissue sites, with the recommendations from BEIR VII [12] for thyroid and breast cancer. UNSCEAR employed Poisson maximum-likelihood methods and Bayesian analysis to represent dosimetry errors to fit generalized ERR and EAR models to the LSS for cancer incidence for REIC. The Hazard rates for cancer mortality *λ_M_* are modeled as recommended by the BEIR VII report [12]. Here λ_MT_ is scaled to λ_IT_ by the ratio of gender, age, and tissue specific mortality to incidence rates from U.S. population data or similar data for NS. Tissue weights and adjustments for never-smokers relative to the U.S. average population assumed in the NSCR-2012 model are given in Cucinotta et al. [5].

**Circulatory Disease REID Estimates**

For circulatory disease risk estimates we use the results of the recent meta-analysis of Little *et al.* [8] for the risk of CVD and IHD. Here a constant ERR/Sv is estimated as 0.21 [0.02, 0.39] for CVD and 0.10 [0.04, 0.15] for IHD. The REID for cancer and circulatory diseases are coupled in the REID evaluation since both will contribute to the survival probability to a given age after exposure.

**Probability of Causation**

The PC or attributable risk is the fraction of the incidence of a disease in a population (exposed and non-exposed) that is due to radiation exposure. Thus the PC represents the incidence of a disease in the population that would be eliminated if there were no radiation exposure. The PC is estimated from Eq. (1) by limiting the upper limit of integration to the date of disease diagnosis, *a_Diag_* for both the exposed population and the reference population, with the PC defined in terms of the conditional tissue specific Excess Relative Risk (ERR) for each tissue:

$$\begin{matrix} (3) & PC & = & \frac{ERR(T,a_{Diag})}{1+ERR(T,a_{Diag})} \end{matrix}$$

where

$$\begin{matrix} (4) & ERR(T,a_{Diag}) & = & \frac{\int_{a_{E}}^{a_{Diag}} dt\lambda_{IT}(a,a_{E},D)S_{0}(t)e^{-\int_{a_{_{E}}}^{t} dz\lambda_{M}(z,a_{E},D)}}{\int_{a_{E}}^{a_{Diag}} dt\lambda_{IT}(a,a_{E},0)S_{0}(t)} & -1 \end{matrix}$$

**Space Radiation Organ Dose Equivalent**For calculations of space radiation tissue specific cancer risks, Eq. (2) is used for the cancer incidence risk rate with the organ dose equivalent estimated using the NASA GCR radiation transport codes, and Badhwar-O’Neill GCR environment [5,14,15]. For GCR the use of risk assessment quantities based on absorbed dose and LET is expected to have short-comings and instead we derived radiation quality descriptors of biological effectiveness based on particle track structure and fluence that were then expressed as radiation quality factors. The NASA quality factor depends on both particle charge number, Z and kinetic energy, E. A key parameter that describes the density of a particle track is Z^*2^/ß^2^ where Z* is the effective charge of a particle and β the particle velocity scaled to the speed of light. In the NASA approach distinct quality factors for estimating solid cancer and leukemia risk are used, Q_solid_ and Q_leukemia_, respectively.

Here a cancer risk cross section representing the biological effect probability per particle is written as [5]:

$$\begin{matrix} (5) & \Sigma(Z,E) & = & \Sigma_{0}[P(Z,E)+\frac{\alpha_{\gamma}}{\Sigma_{0}} \end{matrix}(1-P(Z,E))L]$$

with

where the three parameters of the model ($\Sigma_{0}/\alpha_{\gamma}$, m, and *κ*) based on subjective estimates of results from radiobiology experiments [5]. A radiation quality factor function is then found as:

$$\begin{matrix} (7) & Q_{NASA} & = & (1-P(E,Z))+\frac{6.24(\Sigma_{0}/\alpha_{\gamma})P(E,Z)}{LET} \end{matrix}$$

For calculations for a specific GCR particle type described by *Z* and *E*, Eq. (2) is replaced by

$$\begin{matrix} (8) & \lambda_{ZI}(F_{T},a_{E},a) & = & \lambda_{\gamma I}(a_{E},a)\left\{D_{T}(E,Z)(1-P(Z,E))+(\Sigma_{0}/\alpha_{\gamma})P(Z,E)F_{T}(Z,E)\right\} \end{matrix}$$

where λ_γI_ is the inner bracketed terms in Eq. (2) that contains the ERR and EAR functions for individual tissues. Calculations are made using the NASA models of the GCR environments and radiation transport in spacecraft materials and tissue, which estimate the particle energy spectra,*φ_j_(E)* for 190 isotopes of the elements from Z=1 to 28, neutrons, and contributions pions, electrons and gamma-rays. The fluence spectra, *F(X_tr_)* where X_tr_= Z^*2^/β^2^ can be found by transforming the energy spectra, *φ_j_(E)* for each particle, *j* of mass number and charge number, *A_j_* and *Z_j_* respectively as:

$$\begin{matrix} (9) & F(X_{tr}) & = & \sum_{j} \left(\frac{\partial X_{tr}}{\partial E}\right) \end{matrix}^{-1}\varphi_{j}(E)$$

where we evaluate the Jacobian in Eq. (9) using the Barkas [S1] form for the effective charge number given by

$$\begin{matrix} (10) & Z^{*} & = & Z(1-e^{-125\beta/Z^{2/3}}) \end{matrix}$$

The tissue specific cancer incidence rate for GCR or SPEs can then be written:

$$\begin{matrix} (11) & \lambda_{IT} & \approx& \lambda_{I\gamma}\left\{\sum_{j} \int dE\varphi_{jT}(E)S_{j}(E)(1-P(X_{tr}))+(\Sigma_{0}/\alpha_{\gamma})\int dX_{tr}F(X_{tr})P(X_{tr})\right\} \end{matrix}$$

A summation over all cancer types is made for the radiation contribution to the survivor function in evaluating tissue specific risks, and a further summation over all cancer types to evaluate the over-all cancer risk. Supplementary Fig 1.1 (panel B) illustrates the distribution in solid cancer risk with Z^*2^/β^2^. Pronounced peaks occur as successive values of Z^2^ where β->1 corresponding to the relativistic particle contribution of each GCR charge group.

For circulatory disease risk estimates, information on RBE’s for protons and HZE particles and secondary radiation are even more sparse than those related to cancer risks. For our central estimates we use the RBE’s recommended by the ICRP and NCRP for non-cancer effects with the organ dose equivalent for non-cancer effects, G represented in units of Gy-Eq, G [20,21]. A DDREF is not applied for circulatory disease risks because the meta-analysis of Little *et al*. [8] is based to a large extent on low dose-rate (chronic) exposures to radiation workers, which were fitted with a linear dose response model. We considered several choices for the tissue shielding for the circulatory system, including doses to the blood forming system (BFO), heart, or brain. Because the GCR doses show small variation between tissues (Table S1), these possible choices led to very similar results and we used the BFO average dose, G for non-cancer endpoints to estimate circulatory disease risks.

**Uncertainty Analysis**

To propagate uncertainties across multiple contributors we performed Monte-Carlo simulations sampling over subjective probability distribution functions (PDF) that represent current knowledge of factors that enter into risk models. In a simplified manner, we can write a risk equation as a product of several factors including the dose, *D*, quality factor, *Q*, a low LET risk coefficient normally derived from the data of the atomic-bomb survivors, *R_0_*, and the dose and dose-rate reduction effectiveness factor, *DDREF*, that corrects risk data for dose-rate modifiers. Monte-Carlo uncertainty analysis uses the risk equation, but modified by normal deviates that represent subjective weights and ranges of values for various factors that enter into a risk calculation. First, we define X*∈R(x)* as a random variate that takes on quantiles *x_1_, x_2_, …, x_n_* such that *p(x_i_) =P(X=x_i_)* with the normalization condition *Σp(x_i_)=1*. *C(x_i_)* is defined as the cumulative distribution function, *C(x),* which maps *X* into the uniform distribution *U(0,1)* and we define the inverse cumulative distribution function *C(x)^-1^* in order to perform the inverse mapping of *U(0,1)* into *x*: *x=C(x)^-1^*. Then for a simplified form of the risk equation for a Monte-Carlo trial, *ξ*:

where *R_0_* is the low LET risk coefficient per unit dose, the absorbed dose, *D* is written as the product of the particle fluence, *F* and LET, *L*, and Q the radiation quality factor. The *x_R_, x_phys_, x_Dr_, and x_Q_* are quantiles that represent the uncertainties in the low LET risk coefficient, the space physics models of organ exposures, dose-rate effects, and radiation quality effects, respectively. Monte-Carlo trials are repeated many times, and resulting values binned to form an overall probability distribution function (PDF) taken into account each of the model uncertainties. In practice, the risk model does not use the simple form of Eq.(14). Instead risk calculations are made using the REIC or REID described by Eq. (1). For the 95% Confidence intervals for the %PC, we use the bootstrap method to infer the values from the uncertainty analysis for REIC or REID. PDF functions describing the uncertainties to the quantiles, *ξ_ξ_* for the various parameters in the model are described in Supplementary Table S2. Other details on uncertainty analysis are as described previously [5]. Of note is that using the BEIR VII central estimate of the DDREF as 1.5, leads to a DDREF uncertainty that tends to lower REID estimates, while the uncertainty in the QF tend to increase REID estimates [5].

For circulatory disease risk uncertainties we used a normal distribution to represent the uncertainty that results from the meta-analysis results from Little *et al*. [8] and included the uncertainties in the organ dose estimate for space radiation in the same manner as for cancer risks. For the uncertainty in the RBE for circulatory diseases, we considered the ratio of the organ dose equivalents in Sv, H_T_ to that in Gy-Eq, G_T_ as a reasonable upper estimate in the tissue averaged RBE averaged for non-cancer effects based on known mechanisms for circulatory disease risks from radiation. This ratio varied between 1.7 and 1.9 for typical different spacecraft shielding amounts. Based on these observations, we used a log-normal distribution with GM=1 and GSD=1.35 to represent the PDF for the uncertainty in the tissue averaged RBE for circulatory disease.

**Supplementary References:**

S1. Barkas, H (1963) Nuclear Research Emulsions. Academic Press Inc., New York. Vol. 1, Chap. 9, p. 371.

**Table S1.** Organ doses for 940 day Mars mission at Average solar minimum with 20 g/cm^2^ aluminum shielding. D is the tissue specific absorbed dose, G is the tissue specific dose for non-cancer effects, and H the tissue specific dose equivalent for cancer risks.

| **Females** | | | | **Males** | | | |
| --- | --- | --- | --- | --- | --- | --- | --- |
| **Tissue** | **D, Gy** | **G, Gy-Eq** | **H, Sv** | **Tissue** | **D, Gy** | **G, Gy-Eq** | **H, Sv** |
| Leukemia | 0.395 | 0.605 | 0.554 | Leukemia | 0.392 | 0.602 | 0.55 |
| Stomach | 0.39 | 0.613 | 0.989 | Stomach | 0.388 | 0.61 | 0.983 |
| Colon | 0.394 | 0.603 | 1.004 | Colon | 0.393 | 0.604 | 0.998 |
| Liver | 0.388 | 0.615 | 0.985 | Liver | 0.389 | 0.607 | 0.987 |
| Bladder | 0.388 | 0.621 | 0.985 | Bladder | 0.387 | 0.607 | 0.98 |
| Lung | 0.395 | 0.623 | 1.006 | Lung | 0.391 | 0.609 | 0.993 |
| Esophagus | 0.391 | 0.606 | 0.995 | Esophagus | 0.39 | 0.61 | 0.99 |
| Oral cavity | 0.401 | 0.606 | 1.027 | Oral cavity | 0.394 | 0.61 | 1.003 |
| Brain-CNS | 0.4 | 0.603 | 1.019 | Brain-CNS | 0.398 | 0.6 | 1.015 |
| Thyroid | 0.401 | 0.608 | 1.027 | Thyroid | 0.394 | 0.605 | 1.003 |
| Skin | 0.397 | 0.622 | 1.018 | Skin | 0.401 | 0.62 | 1.034 |
| Remainder | 0.395 | 0.625 | 1.005 | Remainder | 0.392 | 0.613 | 0.998 |
| Breast | 0.398 | 0.613 | 1.017 | Prostate | 0.392 | 0.607 | 0.998 |
| Ovarian | 0.39 | 0.613 | 0.991 |  |  |  |  |
| Uterian | 0.39 | 0.613 | 0.991 |  |  |  |  |

**Table S2.** Summary of PDF for Uncertainty Components in NASA Model for Cancer Risk^5^. Normal distributions described by mean, M and Standard Deviation, SD. Log-normal distributions described by geometric mean, GM, and Geometric Standard Deviation, GSD.

| **Uncertainty Contribution** | **PDF form for Quantile, *x_j_*** | **Comment** |
| --- | --- | --- |
| ***Low LET Model:*** | | |
| **Statistical Errors** | Normal with SD estimates | See ref. [5] |
| **Statistical Errors for Never-smoker, ERR** | Normal (M=1.0; SD=0.25) | Applied to tissues risks adjusted for NS as described previously [5] |
| **Bias in Incidence data** | Normal (M=1.0; SD = 0.05) | Based on NCRP Report 126 [23] |
| **Dosimetry Errors** | Log-Normal (GM=0.9, GSD=1.3) | UNSCEAR Estimate [11] |
| **Transfer model weights** | Uniform distribution about preferred weight | Ignored for breast and thyroid cancers since using Meta-analysis results [12] |
| **DDREF** | Students t-distribution with central estimate of 1.5 | Based on Bayesian analysis of available data [5] |
| ***Risk Cross Section or Q:*** | | |
| ***Σ_0_/α_γ_*** | Log-normal(GM=0.9; GSD=1.4) | GM<1 assumes existing data is biased to higher values |
| **κ** | Normal(M=1, SD=1/3) | Position of peak estimates suggests variation on sensitivity, target size/ distributed targets |
| ***M*** | Discrete m=[2,2.5,3.,3.5,4] with weights [.15,.2,.4,.2,.05] | Values restricted over (2,4) |
| ***Q at high E, low Z*** | Normal (M=1, SD=0.15) | Uncertainty for low LET particles |
| ***Circulatory Disease:*** | | |
| **IHD Statistical Errors** | Normal (M=1.0; SD=0.5) | Based on Little et al. [8] |
| **CVD Statistical Errors** | Normal (M=1.0; SD=0.25) | Based on Little et al. [8] |
| **Tissue Weighted RBE** | Log-Normal (GM=1.0; GSD=1.35) | Based on ratio of Q_ave_ to RBE_ave_ for GCR estimates |
| ***Physics Uncertainties:*** | | |
| **F(Z^*2^/β^2^) for Z<5** | Normal (M=1.05; SD=1/3) | HZETRN does not account for mesons, e- and γ-rays that are low Charge and high velocity; may underestimate neutron recoils of low charge |
| **F(Z^*2^/β^2^) for Z≥5** | Normal (M=1.0; SD=1/4) | HZETRN accurate at high Z |
